# Dramatic secukinumab-mediated improvements in refractory leprosy-related neuritis via the modulation of T helper 1 (Th1) and T helper 17 (Th17) immune pathways

**DOI:** 10.1590/0037-8682-0336-2021

**Published:** 2021-12-17

**Authors:** Patrícia Shu Kurizky, Jorgeth de Oliveira Carneiro da Motta, Natanael Victor Furtunato Bezerra, Marcia Carolline dos Santos Sousa, Danilo Corazza, Taiana Karla dos Santos Borges, Ciro Martins Gomes

**Affiliations:** 1 Universidade de Brasília, Faculdade de Medicina, Programa de Pós-Graduação em Ciências Médicas, Brasília, DF, Brasil.; 2 Universidade de Brasília, Faculdade de Medicina, Hospital Universitário de Brasília, Brasília, DF, Brasil.; 3 Universidade de Brasília, Núcleo de Medicina Tropical, Programa de Pós-Graduação em Medicina Tropical, Brasília, DF, Brasil.

**Keywords:** Leprosy, Interleukin-17, Neuritis

## Abstract

A 39-year-old woman was diagnosed with relapsed multibacillary leprosy and refractory neuritis. Here, we describe an evident loss of therapeutic effectiveness after the third pulse of corticosteroids, which may be attributed to tachyphylaxis and the posterior modulation of interferon- γ (IFN-γ), tumor necrosis factor- α (TNF-α,) interleukin-17A (IL-17A), and IL-12/23p40 after the induction phase of secukinumab. In this case, plasma cytokine analysis showed that secukinumab induced a reduction in IL-17 concomitant with impressive clinical improvements in the patient’s neural function. Interestingly, secukinumab induced reductions in cytokines related to Th1 responses and earlier stages of the Th17 response, including IL-23/12.

## INTRODUCTION

Leprosy remains prevalent in developing areas, and neuritis is one of the major complications associated with the course of the disease^1^. Neural damage is the result of immunological hypersensitivity and occurs more frequently during type I reactional (TIR) states^1^ ^,^ ^2^. We aimed to describe dramatic improvements in refractory leprosy-related neuritis with the use of secukinumab. The patient provided informed consent, and the study was approved by the Ethics Committee of the Faculty of Medicine of the University of Brasilia (5087215.1.0000.5558/72312117.4.0000.5558).

## CASE REPORT

A previously healthy 39-year-old woman was diagnosed with multibacillary leprosy (borderline lepromatous leprosy) in 2014 via positive smear microscopy showing a 3+ bacilloscopic index. The patient was treated with the standard 12-dose multibacillary multidrug therapy for 12 months as recommended by the World Health Organization. The patient was treated again in 2019 because of an unmanageable TIR state, suggesting the possibility of leprosy recurrence. However, smear microscopy was negative. Although *M. leprae* deoxyribonucleic acid was successfully amplified in 2016, no evidence of molecular resistance to rifampicin, dapsone, or ofloxacin treatment was found, as examined by genetic sequencing analysis of rpoB, folp1, and gyrA mutations. Additionally, the patient underwent bilateral neurolysis of the ulnar, median, and posterior tibial nerves. One dexamethasone and two methylprednisolone pulses were also prescribed. The two methylprednisolone pulses showed an evident lack of effectiveness.

**FIGURE 1:**
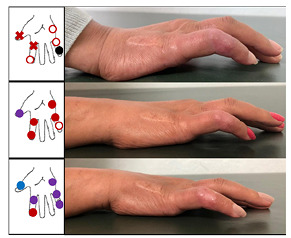
Clinical images showing the improvement in deformities 1 week (upper photograph), 2 weeks (middle photograph) and 1 month (bottom photograph) after the induction phase of secukinumab. The improvements in palmar and plantar tactile sensitivity and related pain were also impressive. Each color represents the lightest tactile sensation felt by esthesiometry (gram-force (gf)): blue circle = 0,2 gf; purple circle = 2,0 gf; full red circle = 4,0 gf; red x = 10 gf; white circle with red edges = 300 gf; black circle = absence of sensitivity.

On November 2020, the patient returned with worsened neuritis and new deformities, despite the daily use of 1 mg/kg prednisone and weekly use of 20 mg methotrexate. Secukinumab was then begun at a dose of 150 mg per week. After induction therapy, a reduction in pain and inflammation was observed. Immunobiological therapy was maintained weekly, following the psoriatic arthritis induction dosing schedule until week 5, after which a monthly dose with consistent reductions in prednisone (from daily 80 mg to daily 20 mg in 5 weeks) was administered, and progressive clinical improvement was observed (Figure 1).

**FIGURE 2:**
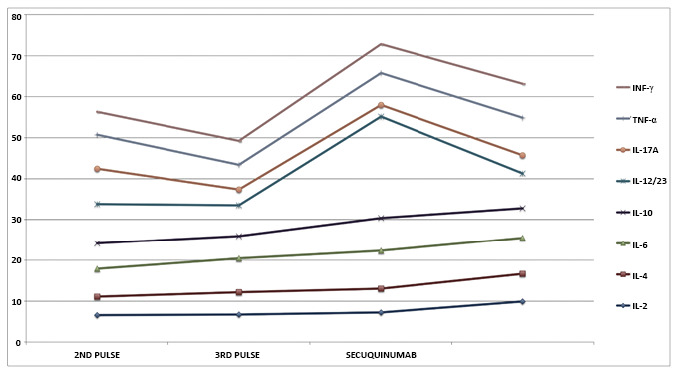
Graph showing basal levels of the measured cytokines after the first corticosteroid pulse and during possible tachyphylaxis. There were reductions in IFN-γ, TNF-α, IL-17A, and IL-12/23p40 levels after the induction phase of secukinumab concomitant with expressive clinical improvements.

Blood was drawn 5 days after each pulse of systemic corticosteroids and after the secukinumab induction phase. Plasma levels of interferon- γ (IFN-γ), tumor necrosis factor- α (TNF-α), interleukin-17A (IL-17A), IL-12/23p40, IL-10, IL-6, IL-4, and IL-2 were measured using the Becton Dickinson (BD) Cytometric Bead Array Human T helper 1 (Th1)/Th2/Th17 Cytokine Kit and the Human IL-12/IL-23p40 Flex Set (RUO) kit on a BD LSR Fortessa™ flow cytometer (BD, Franklyn Lakes, USA) following the manufacturer's instructions. All analyses were performed in triplicate, and values were normalized and compared with those obtained from 10 control and 10 TIR patients. A strong relationship between clinical response and cytokine levels related to Th1 and Th17 immune responses was observed. Figure 2 shows an evident loss of therapeutic effectiveness after the third pulse of corticosteroids, which may be attributed to tachyphylaxis and the posterior modulation of IFN-γ, TNF-α, IL-17A, and IL-12/23p40 after secukinumab induction.

## DISCUSSION

Approximately 40% of multibacillary leprosy patients develop leprosy reactions during the course of disease progression^3^. Signs of nerve injury require immediate treatment to prevent permanent deformity^3^. The role of different T cell subset immune responses is well studied in non-reactional leprosy, but only a few studies have been performed on leprosy reactions^4^ ^,^ ^5^. TIR states are related to a strong Th1 response with autoimmune effects, but other types of responses are also being investigated. Th17 cells are an inflammatory subset of T helper cells described in autoimmune, bacterial, and viral diseases. CD4+ IL-17+ cells participate in immunoregulation and are increased after leprosy treatment^4^. Previous reports showed that IL-17 levels were elevated in the blood of erythema nodosum-affected patients^4^. In addition, the levels of the IL-17F isomer are also elevated in the blood of TIR patients^4^.

In the present case, plasma cytokine analysis showed that secukinumab induced a reduction in IL-17 concomitant with impressive clinical improvements in the patient’s neural function. Interestingly, secukinumab induced reductions in cytokines related to Th1 responses and to earlier stages of the Th17 response, including IL-23/12. It is possible that secukinumab acts as a steroid-sparing agent. Alternatively, as described for psoriasis, blocking IL-17 interrupted the inflammatory stimulus of target cells (e.g., epidermal cells in psoriasis and Schwann cells in leprosy neuritis) and consequently interrupted the attraction of cells related to Th1 and Th17 inflammatory pathways^6^. This connection is described as an inflammatory loop that links different immunological responses^7^. It is also important to state that specific suppression of IL-17 isomers has a smaller effect on immunological protection against mycobacteria in comparison to the use of classical immunosuppressors and TNF-α inhibitors.

In the present report, secukinumab induced a fast and strong reduction in neural inflammation and clinical improvements. The clinical response was consistent with cytokine levels and specific modulation of the Th1 and Th17 response after possible tachyphylaxis in response to corticosteroids. However, there is no validated substitute for corticosteroids in TIR-related neuritis and it causes considerable side effects; thus, further studies are urgently needed to confirm the role of IL-17 inhibitors in leprosy neuritis.
