# Erratum

**DOI:** 10.1590/0037-8682-0336B-2021

**Published:** 2022-04-29

**Revista da Sociedade Brasileira de Medicina Tropical/Journal of the Brazilian Society of Tropical Medicine**

**Title:** Dramatic secukinumab-mediated improvements in refractory leprosy-related neuritis via the modulation of T helper 1 (Th1) and T helper 17 (Th17) immune pathways 

**54: (e0134-2021) 2021 - Page: 1/3 - doi:** 10.1590/0037-8682-0336-2021 

**CORRECT INDEXING OF AUTHOR:**

Taiana Karla dos Santos Borges 

Should read:

 Tatiana Karla dos Santos Borges 

